# Crystal structure and Hirshfeld surface analysis of 2-oxo-13-epi-manoyl oxide isolated from *Sideritis perfoliata*

**DOI:** 10.1107/S2056989018005807

**Published:** 2018-04-27

**Authors:** Ísmail Çelik, Zeliha Atioğlu, Huseyin Aksit, Ibrahim Demirtas, Ramazan Erenler, Mehmet Akkurt

**Affiliations:** a Department of Physics, Faculty of Sciences, Cumhuriyet University, 58140 Sivas, Turkey; b İlke Education and Health Foundation, Cappadocia University, Cappadocia Vocational College, The Medical Imaging Techniques Program, 50420 Mustafapaşa, Ürgüp, Nevşehir, Turkey; c Erzincan University, Faculty of Pharmacy, 24100 Erzincan, Turkey; d Department of Chemistry, Faculty of Natural Sciences, Cankiri Karatekin University, 18100 Cankiri, Turkey; e Department of Chemistry, Faculty of Arts and Sciences, Gaziosmanpasa University, 60240 Tokat, Turkey; f Department of Physics, Faculty of Sciences, Erciyes University, 38039 Kayseri, Turkey

**Keywords:** crystal structure, cyclo­hexane rings, tetra­hydro­pyran rings, helical supra­molecular chains, Hirshfeld surface analysis

## Abstract

The title compound, C_20_H_32_O_2_ (systematic name: 3-ethenyl-3,4a,7,7,10a-penta­methyl­dodeca­hydro-9*H*-benzo[*f*]chromen-9-one), was isolated from *Sideritis perfoliata*. In the crystal, mol­ecules pack in helical supra­molecular chains along the 2_1_ screw axis running parallel to the *a* axis, bound by C—H⋯O hydrogen bonds. These chains are efficiently inter­locked in the other two unit-cell directions *via* van der Waals inter­actions. Hirshfeld surface analysis shows that van der Waals inter­actions constitute the major contribution to the inter­molecular inter­actions, with H⋯H contacts accounting for 86.0% of the surface.

## Abstract 2

In the crystal of the title compound, mol­ecules are linked by C—H⋯O hydrogen bonds, forming *C*(11) helical supra­molecular chains along the 2_1_ axis running parallel to [100].

## Chemical context   

The genus *Sideritis* belonging to the Lamiaceae family is represented by more than 150 species, distributed in tropical regions. Most of the species are found in the Mediterranean region. This genus is represented by 54 species in Turkey flora, 40 of which are endemic (Davis, 1982 ▸). *Sideritis* species have traditionally been used as herbal teas, flavouring agents and therapeutics (Danesi *et al.*, 2013 ▸). *Sideritis* species include flavonoids, terpenes, iridoids, coumarins, lignanes and sterols that are responsible constituents for their pharmacological properties (González-Burgos *et al.*, 2011 ▸). *Sideritis* species have been reported to exhibit considerable biological activities such as anti­oxidant (Demirtas *et al.*, 2011 ▸), anti­proliferative (Demirtas *et al.*, 2009 ▸), and anti­microbial (Yiğit Hanoğlu *et al.*, 2017 ▸) effects. The crystal structure of 2-*β*-hy­droxy­manoyl oxide isolated from *Sideritis perfoliata* has been reported on by our group (Çelik *et al.*, 2016 ▸). Herein, we report on the crystal structure of 2-oxo-13-*epi*-manoyl oxide, also isolated from *S. perfoliata.* 
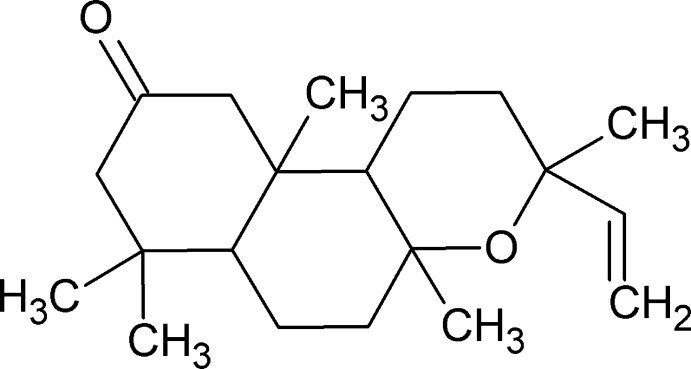


## Structural commentary   

**Figure 1:**
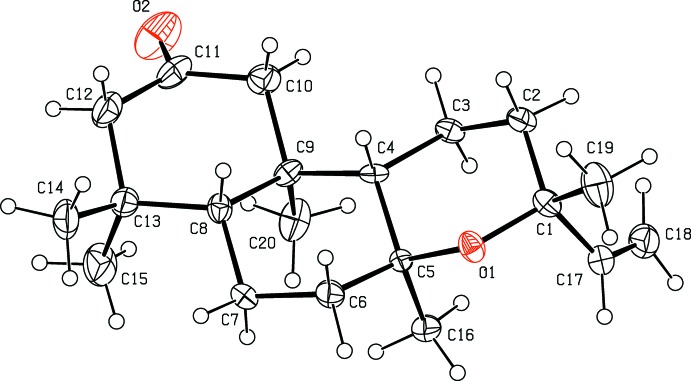
The mol­ecular structure of the title compound, showing the atom labelling and displacement ellipsoids drawn at the 30% probability level.

As shown in Fig. 1 ▸, the junction between the two cyclo­hexane rings *A* (C8–C13) and *B* (C4–C9) is *trans*, and the junction for the tetra­hydro­pyran ring C (O1/C1–C5) is also *trans*. The six-membered carbon rings *A* and *B* possess chair conformations [puckering parameters: *Q*_T_ = 0.528 (7) Å, θ = 172.6 (8)°, φ = 255 (6)° for ring *A* and *Q*_T_ = 0.578 (6) Å, θ = 2.1 (6)°, φ = 261 (16)° for ring *B*]. The tetra­hydro­pyran ring has a slightly twisted boat conformation [puckering parameters: *Q*(2) = 0.411 (6) Å and φ(2) = 81.4 (8)°].

## Supra­molecular features   

**Figure 2:**
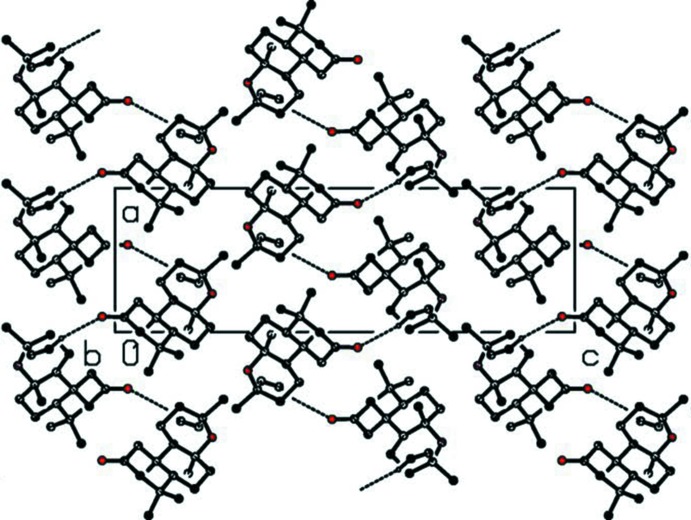
A view along the *a* axis of the crystal packing of the title compound. H atoms not involved in these inter­actions have been omitted for clarity.

In the crystal, mol­ecules pack in helical supra­molecular *C*(11) chains along the 2_1_ screw axis running parallel to the *a* axis, bound by C—H⋯O hydrogen bonds (Fig. 2 ▸ and Table 1 ▸). The chains are efficiently inter­locked in the other two unit-cell directions *via* van der Waals inter­actions. Between the chains there are narrow channels which also run along the [100] direction.

## Database survey   

A search of the Cambridge Structural Database (CSD, V5.39, last update February 2018; Groom *et al.*, 2016 ▸), for 3-ethenyl-3-methyl­dodeca­hydro-1*H*-naphtho­[2,1-*b*]pyran structures, gave 28 hits, all of which present the same basic structural motif as described herein for the title compound. The closest related compound is 2-*β*-hy­droxy­manoyl oxide [systematic name: 3,4a,7,7,10a-penta­methyl-3-vinyl­dodeca­hydro-1*H*-benzo[*f*]chromen- 9-ol] also isolated from *Sideritis perfoliata* (UVEVOI; Çelik *et al.*, 2016 ▸ *).* Other compounds include, Forskolin G (systematic name: 1α-hy­droxy-6β,7β-diacet­oxy-8,13-ep­oxy­labd-14-en-11-one; CSD refcode ADATUV; Shan *et al.*, 2006 ▸), lα,5β-di­hydroxy­manoyl oxide, a novel diterpene from *Satureja gilliesii* (RASXUE; Manríquez *et al.*, 1997 ▸), 4a-hy­droxy-18-normanoyl oxide (GAPZUT; Ybarra *et al.*, 2005 ▸), jhanol (GAQBAC; Ybarra *et al.*, 2005 ▸), 1*R*,11*S*-dihy­droxy-8*R*,13*R*-ep­oxy­labd-14-ene (LUDTOU; Stavri *et al.*, 2009 ▸) and (−)-paniculatol (NEJHAL; Briand *et al.*, 1997 ▸).

In the title compound (*P*2_1_2_1_2_1_, *Z* = 4), the mol­ecules pack in helical supra­molecular chains along the 2_1_ screw axis running parallel to the *a* axis, bound by one C—H⋯O hydrogen bond. These chains are efficiently inter­locked in the other two unit-cell directions *via* van der Waals inter­actions. In the similar compound UVEVOI (*P*2_1_2_1_2_1_, *Z* = 8), the asymmetric unit contains two independent mol­ecules. Inter­molecular O—H⋯O hydrogen bonds connect adjacent mol­ecules, forming *C*(6) helical chains located around a 2_1_ screw axis running along the *a-*axis direction. The crystal packing of these chains is governed only by van der Waals inter­actions. The two asymmetric mol­ecules lead to pseudo-4_1_ symmetry in space group *P*2_1_2_1_2_1_. The crystal structure of the other similar compound UDATUV (*P*2_1_, *Z* = 4) is stabilized by inter­molecular O—H⋯O and C—H⋯O hydrogen bonds, which link the mol­ecules into networks approximately parallel to the (110) plane. In the crystal structure of the compound RASXUE (*P*2_1_, *Z* = 4), no inter­molecular hydrogen-bonding inter­actions were detected, but the O—H⋯O or C—H⋯O inter­actions are possible hydrogen bonds. In GAPZUT (*P*2_1_, *Z* = 6), there are three independent mol­ecules in the asymmetric unit. In the crystal, there is no classical hydrogen bonding·The mol­ecular packing is stabilized by van der Waals inter­actions and no π–π or C—H⋯π inter­actions are observed. In GAQBAC (*P*2_1_, *Z* = 2), mol­ecules are connected by O—H⋯O hydrogen bonds into chains propagating along the *c*-axis direction. Here too, no π–π or C—H⋯π inter­actions are observed. In LUDTOU (*P*2_1_, *Z* = 4), the structure contains a water mol­ecule. In the crystal, mol­ecules are connected *via* O—H⋯O hydrogen bonds involving the water mol­ecules, forming a three-dimensional framework. Again no π–π or C—H⋯π inter­actions are observed.

## Hirshfeld surface analysis   

A large range of properties of inter­molecular close contacts of a structure can be visualized on the Hirshfeld surface with the program *CrystalExplorer* (Wolff *et al.*, 2012 ▸), including *d*_e_ and *d*_i_, which represent the distances from a point on the Hirshfeld surface to the nearest atoms outside (external) and inside (inter­nal) the surface, respectively.

Inter­molecular distance information on the surface can be condensed into a two-dimensional histogram of *d*_e_ and *d*_i_, which is a unique identifier for mol­ecules in a crystal structure, and is known as a fingerprint plot (Rohl *et al.*, 2008 ▸). Instead of plotting *d*_e_ and *d*_i_ on the Hirshfeld surface, contact distances are normalized in *CrystalExplorer* using the van der Waals radius of the appropriate inter­nal (*r*_i_^vdw^) and external (*r*_e_^vdw^) atom of the surface:

*d*_norm_= (*d*_i_-*r*_i_^vdw^)/*r*_i_^vdw^+(*d*_e_-*r*_e_^vdw^)/*r_e_* ^vdw^.

**Figure 3:**
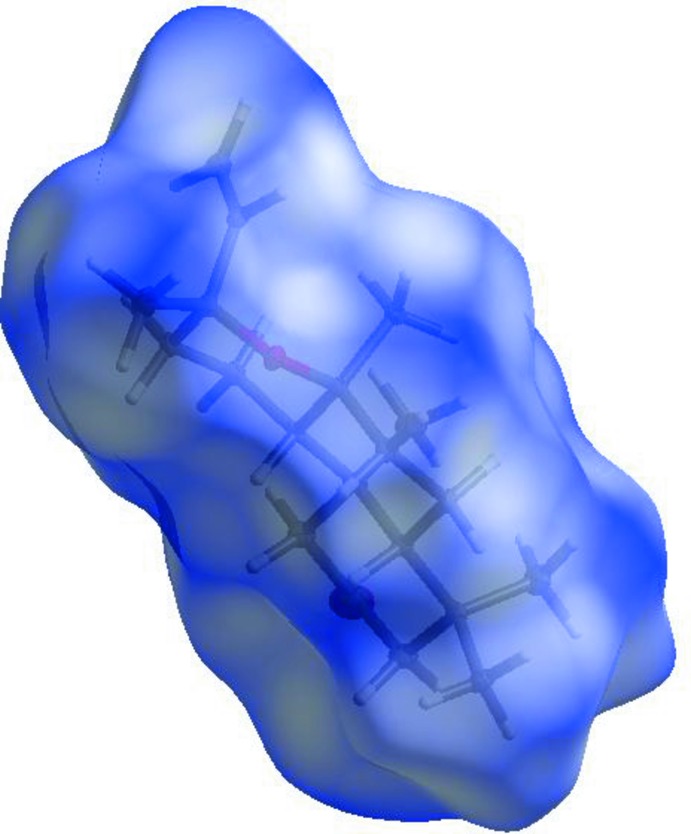
View of the three-dimensional Hirshfeld surface of the title compound mapped with *d*_norm_.

**Figure 4:**
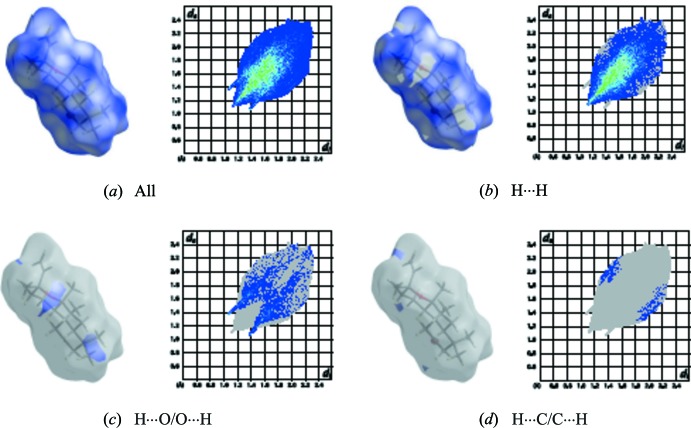
The two-dimensional fingerprint plots of the title compound, showing (*a*) all inter­actions, and delineated into (*b*) H⋯H, (*c*) H⋯O and (*d*) H⋯C inter­actions [*d*_e_ and *d*_i_ represent the distances from a point on the Hirshfeld surface to the nearest atoms outside (external) and inside (inter­nal) the surface, respectively].

**Figure 5:**
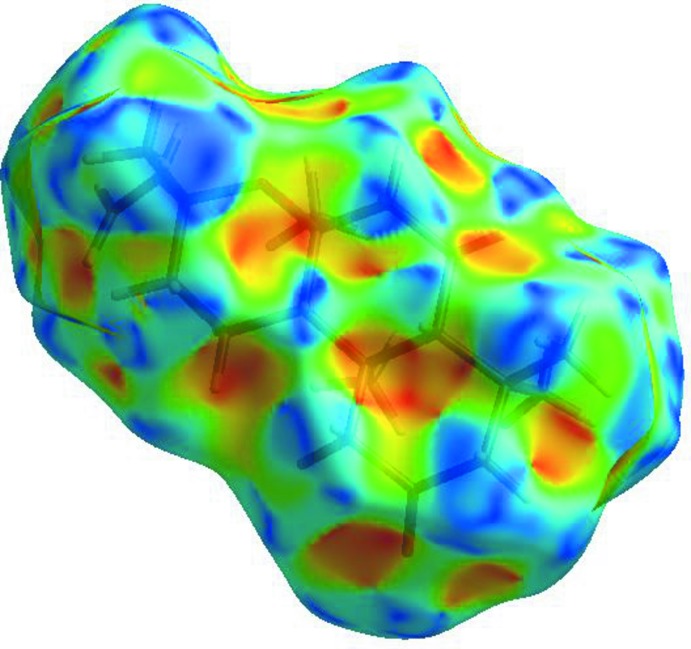
Hirshfeld surface of the title complex plotted over the shape-index.

For the title compound, the three-dimensional Hirshfeld surface mapped over *d_norm_* is given in Fig. 3 ▸. Contacts with distances equal to the sum of the van der Waals radii are shown in white, and contacts with distances shorter than or longer than the related sum values are shown in red (highlighted contacts) or blue, respectively. Two-dimensional finger print plots showing the occurrence of the various inter­molecular contacts are presented in Fig. 4 ▸ *a*–*d*. The H⋯H inter­actions appear in the middle of the scattered points in the two-dimensional fingerprint plots with an overall contribution to the Hirshfeld surface of 86.0% (Fig. 4 ▸ *b*). The contribution from the H⋯O/O⋯H contacts, corresponding to C—H⋯O inter­actions, is represented by a pair of sharp spikes characteristic of a strong hydrogen-bond inter­action (12.6%) (Fig. 4 ▸ *c*). The contribution of the other inter­molecular contacts to the Hirshfeld surfaces is H⋯C/C⋯H (1.4%) (Fig. 4 ▸ *d*). The large number of H⋯H, H⋯O/O⋯H and H⋯C/C⋯H inter­actions suggest that van der Waals inter­actions and hydrogen bonding play the major roles in the crystal packing (Hathwar *et al.*, 2015 ▸). A view of the Hirshfeld surface of the title complex plotted over the shape-index is given in Fig. 5 ▸.

## Synthesis and crystallization   

The floral parts of *Sideritis perfoliata* (100 g) were extracted with EtOAc (3 × 1.0 L). After removal of the solvent *in vacuo*, the extract (4.0 g) was subjected to Sephadex LH-20 column chromatography using methanol as the mobile phase at 0.5 ml/min flow rate. According to TLC basis the 6–8th fractions were combined (1.2 g) and separated over silica gel column chromatography using a hexa­ne/EtOAc (6/4) mixture. Fractions 2–4 were combined to give 2-*oxo*-13-*epi*-manoyl oxide (60 mg). After removal of the solvent, a white amorphous powder was obtained. The solid was dissolved in acetone and left to stand at room temperature for 12 h. On slow evaporation of the solvent, colourless block-like crystals were obtained.

## Refinement details   

Crystal data, data collection and structure refinement details are summarized in Table 2 ▸. All H atoms were placed in geometrically idealized positions and constrained to ride on their parent atoms: C—H = 0.93–0.97 Å with *U*_iso_(H) = 1.5*U*_eq_(C-meth­yl) and 1.2*U*_eq_(C) for other H atoms. As the title compound is a weak anomalous scatterer, the value of the Flack parameter of −1.1 (10) is meaningless.

## Supplementary Material

Crystal structure: contains datablock(s) global, I. DOI: 10.1107/S2056989018005807/su5431sup1.cif

Structure factors: contains datablock(s) I. DOI: 10.1107/S2056989018005807/su5431Isup2.hkl

Supporting information file. DOI: 10.1107/S2056989018005807/su5431Isup3.cml

CCDC reference: 1837011

Additional supporting information:  crystallographic information; 3D view; checkCIF report

## Figures and Tables

**Table 1:** Hydrogen-bond geometry (Å, °)

*D*—H⋯*A*	*D*—H	H⋯*A*	*D*⋯*A*	*D*—H⋯*A*
C18—H18*A*⋯O2^i^	0.93	2.59	3.501 (9)	167

Symmetry code: (i) 
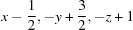
.

**Table 2:** Experimental details

Crystal data	
Chemical formula	C_20_H_32_O_2_
*M*_r_	304.46
Crystal system, space group	Orthorhombic, *P*2_1_2_1_2_1_
Temperature (K)	296
*a*, *b*, *c* (Å)	7.803 (2), 9.242 (3), 24.952 (7)
*V* (Å^3^)	1799.4 (9)
*Z*	4
Radiation type	Mo *K*α
μ (mm^−1^)	0.07
Crystal size (mm)	0.12 × 0.11 × 0.09

Data collection	
Diffractometer	Bruker APEXII CCD
Absorption correction	Multi-scan (*SADABS*; Bruker, 2007 ▸)
*T*_min_, *T*_max_	0.596, 0.745
No. of measured, independent and observed [*I* > 2σ(*I*)] reflections	11666, 3530, 2120
*R*_int_	0.097
(sin θ/λ)_max_ (Å^−1^)	0.626

Refinement	
*R*[*F* ^2^ > 2σ(*F* ^2^)], *wR*(*F* ^2^), *S*	0.096, 0.186, 1.27
No. of reflections	3530
No. of parameters	204
H-atom treatment	H-atom parameters constrained
Δρ_max_, Δρ_min_ (e Å^−3^)	0.21, −0.25

Computer programs: *APEX2* and *SAINT* (Bruker, 2007 ▸), *SHELXS97* (Sheldrick, 2008 ▸), *SHELXL2014* (Sheldrick, 2015 ▸), *ORTEP-3 for Windows* (Farrugia, 2012 ▸) and *PLATON* (Spek, 2009 ▸).

## supplementary crystallographic information

### Crystal data

**Table:**

C_20_H_32_O_2_	*F*(000) = 672
*M**_r_* = 304.46	*D*_x_ = 1.124 Mg m^−^^3^
Orthorhombic, *P*2_1_2_1_2_1_	Mo *K*α radiation, λ = 0.71073 Å
Hall symbol: P 2ac 2ab	Cell parameters from 6426 reflections
*a* = 7.803 (2) Å	θ = 3.1–26.4°
*b* = 9.242 (3) Å	µ = 0.07 mm^−^^1^
*c* = 24.952 (7) Å	*T* = 296 K
*V* = 1799.4 (9) Å^3^	Block, colourless
*Z* = 4	0.12 × 0.11 × 0.09 mm

### Data collection

**Table:**

Bruker APEXII CCD diffractometer	2120 reflections with *I* > 2σ(*I*)
φ and ω scans	*R*_int_ = 0.097
Absorption correction: multi-scan (SADABS; Bruker, 2007)	θ_max_ = 26.4°, θ_min_ = 3.1°
*T*_min_ = 0.596, *T*_max_ = 0.745	*h* = −8→9
11666 measured reflections	*k* = −11→11
3530 independent reflections	*l* = −30→31

### Refinement

**Table:**

Refinement on *F*^2^	Primary atom site location: structure-invariant direct methods
Least-squares matrix: full	Secondary atom site location: difference Fourier map
*R*[*F*^2^ > 2σ(*F*^2^)] = 0.096	Hydrogen site location: inferred from neighbouring sites
*wR*(*F*^2^) = 0.186	H-atom parameters constrained
*S* = 1.27	*w* = 1/[σ^2^(*F*_o_^2^) + (0.0079*P*)^2^ + 2.0609*P*] where *P* = (*F*_o_^2^ + 2*F*_c_^2^)/3
3530 reflections	(Δ/σ)_max_ < 0.001
204 parameters	Δρ_max_ = 0.21 e Å^−^^3^
0 restraints	Δρ_min_ = −0.25 e Å^−^^3^

### Special details

**Table:**

Geometry. Bond distances, angles etc. have been calculated using the rounded fractional coordinates. All su's are estimated from the variances of the (full) variance-covariance matrix. The cell esds are taken into account in the estimation of distances, angles and torsion angles
Refinement. Refinement on F^2^ for ALL reflections except those flagged by the user for potential systematic errors. Weighted R-factors wR and all goodnesses of fit S are based on F^2^, conventional R-factors R are based on F, with F set to zero for negative F^2^. The observed criterion of F^2^ > 2sigma(F^2^) is used only for calculating -R-factor-obs etc. and is not relevant to the choice of reflections for refinement. R-factors based on F^2^ are statistically about twice as large as those based on F, and R-factors based on ALL data will be even larger.

### Fractional atomic coordinates and isotropic or equivalent isotropic displacement parameters (Å^2^)

**Table:**

	*x*	*y*	*z*	*U*_iso_*/*U*_eq_	
O1	0.2335 (5)	0.8443 (5)	0.71085 (15)	0.0343 (16)	
O2	0.3930 (9)	0.4214 (6)	0.47206 (19)	0.083 (3)	
C1	0.0977 (8)	0.9360 (7)	0.6904 (2)	0.034 (2)	
C2	0.0185 (8)	0.8669 (7)	0.6406 (2)	0.037 (2)	
C3	0.1511 (8)	0.8173 (7)	0.5996 (2)	0.034 (2)	
C4	0.2784 (8)	0.7152 (6)	0.6273 (2)	0.0247 (19)	
C5	0.3660 (8)	0.7939 (6)	0.6745 (2)	0.0257 (19)	
C6	0.4657 (8)	0.6837 (7)	0.7072 (2)	0.036 (2)	
C7	0.5901 (8)	0.5959 (7)	0.6731 (2)	0.035 (2)	
C8	0.4971 (9)	0.5188 (7)	0.6269 (2)	0.0310 (19)	
C9	0.4001 (9)	0.6277 (6)	0.5902 (2)	0.0303 (19)	
C10	0.2898 (10)	0.5375 (8)	0.5505 (2)	0.044 (3)	
C11	0.3961 (11)	0.4285 (8)	0.5206 (3)	0.050 (3)	
C12	0.4981 (10)	0.3273 (7)	0.5547 (3)	0.051 (3)	
C13	0.6085 (9)	0.4010 (7)	0.5985 (3)	0.038 (2)	
C14	0.6582 (10)	0.2831 (8)	0.6390 (3)	0.056 (3)	
C15	0.7747 (11)	0.4588 (9)	0.5732 (3)	0.067 (3)	
C16	0.4820 (9)	0.9208 (7)	0.6596 (3)	0.040 (2)	
C17	0.1589 (9)	1.0879 (7)	0.6821 (3)	0.039 (2)	
C18	0.1195 (10)	1.1772 (8)	0.6434 (3)	0.050 (3)	
C19	−0.0318 (10)	0.9399 (8)	0.7360 (3)	0.059 (3)	
C20	0.5154 (11)	0.7268 (7)	0.5559 (3)	0.049 (3)	
H2A	−0.04990	0.78430	0.65140	0.0440*	
H2B	−0.05760	0.93630	0.62370	0.0440*	
H3A	0.21130	0.90030	0.58510	0.0410*	
H3B	0.09470	0.76740	0.57030	0.0410*	
H4	0.20610	0.64180	0.64440	0.0300*	
H6A	0.38570	0.61840	0.72450	0.0430*	
H6B	0.52920	0.73360	0.73500	0.0430*	
H7A	0.64680	0.52460	0.69540	0.0420*	
H7B	0.67720	0.65970	0.65850	0.0420*	
H8	0.40580	0.46380	0.64450	0.0370*	
H10A	0.20050	0.48750	0.57030	0.0530*	
H10B	0.23500	0.60210	0.52520	0.0530*	
H12A	0.57310	0.27130	0.53160	0.0610*	
H12B	0.41990	0.26020	0.57190	0.0610*	
H14A	0.55750	0.25030	0.65750	0.0840*	
H14B	0.73820	0.32210	0.66440	0.0840*	
H14C	0.70990	0.20330	0.62040	0.0840*	
H15A	0.83750	0.51340	0.59950	0.1000*	
H15B	0.74710	0.52010	0.54340	0.1000*	
H15C	0.84340	0.37910	0.56110	0.1000*	
H16A	0.42730	0.97830	0.63240	0.0600*	
H16B	0.58910	0.88470	0.64610	0.0600*	
H16C	0.50240	0.97930	0.69070	0.0600*	
H17	0.23470	1.12310	0.70770	0.0470*	
H18A	0.04430	1.14840	0.61660	0.0600*	
H18B	0.16670	1.26960	0.64280	0.0600*	
H19A	−0.07240	0.84370	0.74300	0.0890*	
H19B	−0.12660	1.00050	0.72610	0.0890*	
H19C	0.02180	0.97810	0.76760	0.0890*	
H20A	0.44920	0.80690	0.54280	0.0740*	
H20B	0.56040	0.67290	0.52620	0.0740*	
H20C	0.60830	0.76240	0.57740	0.0740*	

### Atomic displacement parameters (Å^2^)

**Table:**

	*U*^11^	*U*^22^	*U*^33^	*U*^12^	*U*^13^	*U*^23^
O1	0.032 (3)	0.044 (3)	0.027 (2)	0.010 (2)	0.0025 (19)	0.000 (2)
O2	0.132 (6)	0.082 (4)	0.035 (3)	−0.004 (4)	0.005 (3)	−0.019 (3)
C1	0.025 (4)	0.040 (4)	0.038 (3)	0.001 (3)	0.002 (3)	0.001 (3)
C2	0.025 (4)	0.039 (4)	0.047 (4)	−0.002 (3)	−0.005 (3)	0.000 (3)
C3	0.032 (4)	0.040 (4)	0.030 (3)	0.003 (3)	−0.014 (3)	0.000 (3)
C4	0.022 (3)	0.031 (4)	0.021 (3)	−0.010 (3)	−0.002 (3)	0.000 (3)
C5	0.023 (3)	0.027 (4)	0.027 (3)	0.002 (3)	−0.003 (3)	−0.006 (3)
C6	0.039 (4)	0.041 (4)	0.028 (3)	0.007 (4)	−0.011 (3)	−0.003 (3)
C7	0.025 (4)	0.038 (4)	0.043 (4)	0.004 (3)	−0.007 (3)	−0.003 (3)
C8	0.030 (4)	0.030 (3)	0.033 (3)	0.003 (3)	0.008 (3)	0.000 (3)
C9	0.041 (4)	0.028 (3)	0.022 (3)	−0.005 (3)	0.001 (3)	−0.002 (3)
C10	0.051 (5)	0.046 (5)	0.035 (4)	0.000 (4)	−0.010 (3)	−0.002 (3)
C11	0.066 (5)	0.042 (4)	0.042 (4)	−0.010 (4)	0.004 (4)	−0.017 (4)
C12	0.058 (5)	0.040 (4)	0.054 (4)	0.000 (4)	0.010 (4)	−0.016 (4)
C13	0.033 (4)	0.033 (4)	0.049 (4)	−0.003 (3)	0.007 (3)	−0.004 (3)
C14	0.056 (5)	0.037 (4)	0.076 (6)	0.016 (4)	0.004 (4)	−0.002 (4)
C15	0.052 (5)	0.063 (6)	0.086 (6)	0.004 (5)	0.029 (5)	−0.011 (5)
C16	0.031 (4)	0.035 (4)	0.055 (4)	−0.005 (4)	0.000 (3)	−0.007 (3)
C17	0.034 (4)	0.035 (4)	0.048 (4)	0.006 (3)	−0.003 (3)	−0.005 (4)
C18	0.043 (5)	0.039 (4)	0.068 (5)	0.001 (4)	0.004 (4)	0.000 (4)
C19	0.053 (5)	0.068 (5)	0.057 (5)	0.017 (5)	0.024 (4)	0.005 (4)
C20	0.070 (6)	0.040 (4)	0.038 (4)	0.004 (4)	0.024 (4)	0.004 (3)

### Geometric parameters (Å, º)

**Table:**

O1—C1	1.450 (7)	C4—H4	0.9800
O1—C5	1.452 (7)	C6—H6A	0.9700
O2—C11	1.213 (9)	C6—H6B	0.9700
C1—C2	1.528 (8)	C7—H7A	0.9700
C1—C17	1.497 (9)	C7—H7B	0.9700
C1—C19	1.522 (9)	C8—H8	0.9800
C2—C3	1.526 (8)	C10—H10A	0.9700
C3—C4	1.535 (8)	C10—H10B	0.9700
C4—C5	1.544 (8)	C12—H12A	0.9700
C4—C9	1.553 (8)	C12—H12B	0.9700
C5—C6	1.519 (8)	C14—H14A	0.9600
C5—C16	1.527 (9)	C14—H14B	0.9600
C6—C7	1.525 (8)	C14—H14C	0.9600
C7—C8	1.537 (8)	C15—H15A	0.9600
C8—C9	1.557 (8)	C15—H15B	0.9600
C8—C13	1.563 (9)	C15—H15C	0.9600
C9—C10	1.555 (9)	C16—H16A	0.9600
C9—C20	1.543 (10)	C16—H16B	0.9600
C10—C11	1.503 (10)	C16—H16C	0.9600
C11—C12	1.494 (11)	C17—H17	0.9300
C12—C13	1.549 (10)	C18—H18A	0.9300
C13—C14	1.536 (10)	C18—H18B	0.9300
C13—C15	1.538 (11)	C19—H19A	0.9600
C17—C18	1.307 (10)	C19—H19B	0.9600
C2—H2A	0.9700	C19—H19C	0.9600
C2—H2B	0.9700	C20—H20A	0.9600
C3—H3A	0.9700	C20—H20B	0.9600
C3—H3B	0.9700	C20—H20C	0.9600
			
C1—O1—C5	119.2 (4)	C7—C6—H6A	109.00
O1—C1—C2	109.7 (5)	C7—C6—H6B	109.00
O1—C1—C17	111.3 (5)	H6A—C6—H6B	108.00
O1—C1—C19	103.7 (5)	C6—C7—H7A	109.00
C2—C1—C17	114.1 (5)	C6—C7—H7B	109.00
C2—C1—C19	110.5 (5)	C8—C7—H7A	109.00
C17—C1—C19	107.0 (5)	C8—C7—H7B	109.00
C1—C2—C3	113.4 (5)	H7A—C7—H7B	108.00
C2—C3—C4	108.8 (4)	C7—C8—H8	104.00
C3—C4—C5	109.9 (5)	C9—C8—H8	104.00
C3—C4—C9	116.6 (4)	C13—C8—H8	104.00
C5—C4—C9	115.4 (5)	C9—C10—H10A	109.00
O1—C5—C4	108.2 (5)	C9—C10—H10B	109.00
O1—C5—C6	104.1 (4)	C11—C10—H10A	109.00
O1—C5—C16	109.1 (5)	C11—C10—H10B	109.00
C4—C5—C6	108.7 (5)	H10A—C10—H10B	108.00
C4—C5—C16	116.0 (5)	C11—C12—H12A	108.00
C6—C5—C16	110.0 (5)	C11—C12—H12B	108.00
C5—C6—C7	112.5 (4)	C13—C12—H12A	109.00
C6—C7—C8	111.4 (5)	C13—C12—H12B	109.00
C7—C8—C9	111.8 (5)	H12A—C12—H12B	108.00
C7—C8—C13	113.6 (6)	C13—C14—H14A	109.00
C9—C8—C13	117.0 (5)	C13—C14—H14B	109.00
C4—C9—C8	106.5 (4)	C13—C14—H14C	109.00
C4—C9—C10	108.7 (5)	H14A—C14—H14B	109.00
C4—C9—C20	112.2 (5)	H14A—C14—H14C	109.00
C8—C9—C10	107.3 (5)	H14B—C14—H14C	110.00
C8—C9—C20	115.2 (6)	C13—C15—H15A	109.00
C10—C9—C20	106.7 (5)	C13—C15—H15B	110.00
C9—C10—C11	111.7 (6)	C13—C15—H15C	109.00
O2—C11—C10	121.4 (7)	H15A—C15—H15B	109.00
O2—C11—C12	123.1 (7)	H15A—C15—H15C	109.00
C10—C11—C12	115.5 (6)	H15B—C15—H15C	110.00
C11—C12—C13	115.0 (6)	C5—C16—H16A	109.00
C8—C13—C12	108.5 (6)	C5—C16—H16B	110.00
C8—C13—C14	109.7 (6)	C5—C16—H16C	110.00
C8—C13—C15	114.4 (6)	H16A—C16—H16B	109.00
C12—C13—C14	107.0 (5)	H16A—C16—H16C	109.00
C12—C13—C15	109.4 (6)	H16B—C16—H16C	110.00
C14—C13—C15	107.7 (6)	C1—C17—H17	116.00
C1—C17—C18	128.3 (7)	C18—C17—H17	116.00
C1—C2—H2A	109.00	C17—C18—H18A	120.00
C1—C2—H2B	109.00	C17—C18—H18B	120.00
C3—C2—H2A	109.00	H18A—C18—H18B	120.00
C3—C2—H2B	109.00	C1—C19—H19A	109.00
H2A—C2—H2B	108.00	C1—C19—H19B	109.00
C2—C3—H3A	110.00	C1—C19—H19C	109.00
C2—C3—H3B	110.00	H19A—C19—H19B	109.00
C4—C3—H3A	110.00	H19A—C19—H19C	110.00
C4—C3—H3B	110.00	H19B—C19—H19C	109.00
H3A—C3—H3B	108.00	C9—C20—H20A	109.00
C3—C4—H4	104.00	C9—C20—H20B	109.00
C5—C4—H4	104.00	C9—C20—H20C	109.00
C9—C4—H4	105.00	H20A—C20—H20B	110.00
C5—C6—H6A	109.00	H20A—C20—H20C	109.00
C5—C6—H6B	109.00	H20B—C20—H20C	109.00
			
C5—O1—C1—C2	50.8 (7)	C4—C5—C6—C7	53.8 (7)
C5—O1—C1—C17	−76.5 (6)	C16—C5—C6—C7	−74.3 (6)
C5—O1—C1—C19	168.8 (5)	C5—C6—C7—C8	−56.6 (7)
C1—O1—C5—C6	−171.1 (5)	C6—C7—C8—C9	57.8 (7)
C1—O1—C5—C16	71.5 (6)	C6—C7—C8—C13	−167.1 (5)
C1—O1—C5—C4	−55.5 (6)	C7—C8—C9—C4	−55.5 (6)
C19—C1—C2—C3	−162.4 (5)	C7—C8—C9—C10	−171.7 (5)
C2—C1—C17—C18	17.1 (10)	C7—C8—C9—C20	69.6 (6)
C19—C1—C17—C18	−105.4 (8)	C13—C8—C9—C4	171.1 (5)
O1—C1—C17—C18	142.0 (7)	C13—C8—C9—C10	54.9 (7)
O1—C1—C2—C3	−48.8 (6)	C13—C8—C9—C20	−63.8 (7)
C17—C1—C2—C3	76.9 (7)	C7—C8—C13—C12	177.6 (5)
C1—C2—C3—C4	55.0 (7)	C7—C8—C13—C14	61.0 (7)
C2—C3—C4—C9	167.5 (5)	C7—C8—C13—C15	−60.1 (7)
C2—C3—C4—C5	−58.7 (6)	C9—C8—C13—C12	−49.9 (7)
C3—C4—C5—C6	169.8 (5)	C9—C8—C13—C14	−166.4 (6)
C9—C4—C5—C6	−55.9 (6)	C9—C8—C13—C15	72.5 (8)
C3—C4—C5—C16	−65.7 (7)	C4—C9—C10—C11	−168.9 (5)
C9—C4—C5—O1	−168.4 (4)	C8—C9—C10—C11	−54.1 (7)
C3—C4—C5—O1	57.3 (6)	C20—C9—C10—C11	69.9 (7)
C3—C4—C9—C20	60.6 (7)	C9—C10—C11—O2	−127.6 (8)
C5—C4—C9—C8	56.3 (6)	C9—C10—C11—C12	54.9 (8)
C5—C4—C9—C10	171.6 (5)	O2—C11—C12—C13	132.0 (8)
C5—C4—C9—C20	−70.7 (6)	C10—C11—C12—C13	−50.5 (9)
C9—C4—C5—C16	68.6 (6)	C11—C12—C13—C8	45.0 (8)
C3—C4—C9—C8	−172.5 (5)	C11—C12—C13—C14	163.2 (6)
C3—C4—C9—C10	−57.2 (6)	C11—C12—C13—C15	−80.4 (8)
O1—C5—C6—C7	168.9 (5)		

### Hydrogen-bond geometry (Å, º)

**Table:**

*D*—H···*A*	*D*—H	H···*A*	*D*···*A*	*D*—H···*A*
C18—H18*A*···O2^i^	0.93	2.59	3.501 (9)	167

Symmetry code: (i) *x*−1/2, −*y*+3/2, −*z*+1.
